# Reports of L-Norvaline Toxicity in Humans May Be Greatly Overstated

**DOI:** 10.3390/brainsci9120382

**Published:** 2019-12-17

**Authors:** Baruh Polis, Michael A. Gilinsky, Abraham O. Samson

**Affiliations:** 1 Drug Discovery Laboratory, The Azrieli Faculty of Medicine, Bar-Ilan University, Safed 1311502, Israel; avraham.samson@biu.ac.il; 2 Scientific Research Institute of Physiology and Basic Medicine, 4 Timakova St., Novosibirsk 630117, Russia; m.a.gilinsky@physiol.ru

**Keywords:** L-norvaline, supplement, toxicity, brain

## Abstract

Recently, a study published in “Toxicology In Vitro” (Kate Samardzic and Kenneth J. Rodgers) was entitled: “Cytotoxicity and Mitochondrial Dysfunction Caused by the Dietary Supplement L-Norvaline”. The title may be greatly overstated, and here we provide several arguments showing that norvaline is not as toxic as reported.

Recently, a study published in “Toxicology In Vitro” (Kate Samardzic and Kenneth J. Rodgers 2019) was entitled: “Cytotoxicity and Mitochondrial Dysfunction Caused by the Dietary Supplement l-Norvaline” [1]. The title, however, may be greatly overstated and raises several concerns. 

First, the concentrations of L-norvaline used in the study may reduce cell viability (>125 µM), but not for norvaline itself. In fact, it is well-established that most amino acids at concentrations ~100 µM and above are cytotoxic in vitro. The pioneering studies by Harry Eagle (1955) in human carcinoma HeLa cells have convincingly substantiated the phenomenon known as “the dose makes the poison” [2]. More recently, studies have shown that branched-chain amino acids (BCAA) induce cell senescence through DNA damage. For instance, a study by Raquelda Luz Dias et al. (2018), published earlier in the same journal, demonstrated that osteoblast precursor cells (MC3T3-E1) treated with 50 μM of L-leucine for 48 h—which corresponds just to a 12.5% increase of the amino acid in the medium—decreased proliferation by 40% through mechanisms not related to cell necrosis, apoptosis, but through DNA damage and cell senescence mechanisms [3]. Thus, the title assertion that L-norvaline is cytotoxic needs to be limited only to concentrations higher than 125 µM, which is also true for the vast majority of natural amino acids.

Second, the model used in the study is an immortal cell line (SH-SY5Y human neuroblastoma cells), and not an entire organism. In higher organisms, L-norvaline is well-tolerated, and in vivo toxicity is not apparent [4]. Moreover, treatment with L-norvaline is neuroprotective in a mouse model of Alzheimer’s disease [5]. Additionally, L-norvaline has been shown to possess beneficial anti-inflammatory properties, which are apparent in human endothelial cells at the concentrations of 10–40 mM [6]. Notably, even the authors admit that, in combination with L-valine, L-norvaline is proliferative, and not cytotoxic. In combination with L-leucine and L-isoleucine, the study at hand finds L-norvaline to have little effect on cell viability. Only in combination with L-serine is the anti-proliferative effect of L-norvaline pronounced. Accordingly, the assertion that l-norvaline is cytotoxic needs to be limited only to very specific conditions in vitro.

Third, the mitochondrial dysfunction assay used in the study also raises concerns. The authors utilize L-norvaline at concentrations of 500 and 2000 µM, with and without L-NAME, a nitric oxide synthase inhibitor. Mitochondrial dysfunction is shown to be significant only in the presence of 200 µM L-NAME and at L-norvaline concentrations of 2000 µM. Thus, the assertion that L-norvaline alone leads to mitochondrial dysfunction is unfounded. 

It is noteworthy that L-norvaline is able to be a substrate for branched-chain amino acid aminotransferase (BCAT) [7], which is present in neurons and glial cells. BCAT catalyzes the conversion of branched-chain amino acids and α-ketoglutarate into branched-chain α-keto acids and glutamate. Therefore, the cytotoxic effect of L-norvaline in SH-SY5Y cells might be glutamate- and calcium-mediated. In accordance with our vision of the L-norvaline effects upon glutamate homeostasis in the brain, the agent upsurges the production of glutamate, which is the primary source of cytotoxicity observed in vitro. Astrocytes play a central role in maintaining of glutamate homeostasis in the brain by controlling the balance between glutamate uptake and release [8]. They control and modulate extracellular levels of glutamate via glutamate transporters present in their membrane [9]. Moreover, astrocytes express extensively vesicular glutamate transporters (VGLUT) and are capable of vesicular loading and release of glutamate [10]. We have previously shown that L-norvaline significantly increases (by hundreds of percent) the levels of VGLUT1 and VGLUT3 in the hippocampi of Alzheimer’s disease mice [5]. Thus, we speculate that this mechanism prevents an escalation of extracellular glutamate levels in vivo. For that reason, the cellular models that lack astrocytes may not represent a physiologically relevant system in cytotoxicity experiments which test glutamate-associated toxicity. 

Furthermore, the tests with various toxins/toxicants in co-cultured neuronal–astrocyte cells, even in transwell/restricted contact models, have demonstrated their suitability to better evaluate human neuronal toxicity and neuroprotective effects of emerging drugs. For example, a recent comparative study by De Simone et al. (2017) evidenced a substantial improvement of cell viability (by dozens of percent) in a model of SH-SY5Y cells co-cultured with astrocytes [11]. Remarkably, the examination of three different toxins demonstrated a significant improvement in mitochondrial function and cell morphology in the co-cultured model compared to single cell cultures. Accordingly, co-culture systems provide a more representative central nervous system (CNS) in vivo-like tissue.

In fact, the authors addressed the issue of glutamate toxicity in their work and performed a set of highly relevant assays. Nevertheless, the glutamate content and intracellular Ca^2+^ levels have also been tested in cells treated with extremely high L-norvaline concentrations (2000 µM), which led to misinterpretation. Moreover, the authors deduce that L-norvaline does not influence intracellular calcium concentrations, even though L-norvaline-treated cells show relative calcium-dependent fluorescence, which is two-fold higher than in control cells; therefore, this particular assay may benefit from validation by other methods.

It is worth highlighting that, in order to cover the physiological requirements, the adult organism should receive daily about 25 mg/kg of valine [12], which corresponds to consuming about 2 g of valine every day by an adult person. Furthermore, L-valine concentration in the cerebrospinal fluid (CSF) of healthy individuals is about 20 µM [13], which is substantially lower than cytotoxic concentrations (>125 µM) of L-norvaline tested by the authors. L-norvaline is used by sportsmen in a dose, which varies between 200 and 300 mg/day, about tenfold less than the regular consumption of L-valine. Therefore, assuming the same transport mechanisms via the blood–brain barrier (BBB) and taking into account the transport competition with large neutral amino acids, we suggest that actual concentrations of L-norvaline in athletes are significantly lower than those tested in vitro. This estimation accords with the results published by Jean K. Tews and Alfred E. Harper (1986), and quoted by the authors, establishing the brain concentration of L-norvaline as 0.09 µM/g in rats fed on a low protein diet containing huge amounts (1%) of L-norvaline [14]. Also, the same study demonstrated that analog-induced depression in brain BCAA levels is almost completely alleviated when the diet contains a high protein level. 

In brief, the conclusions of the study by Samardzic and Rodgers are significantly overstated and omit the fact that L-norvaline toxicity is limited to specific in vitro assays at exceedingly high concentrations. As such, the title could inadvertently be grossly exaggerated and may have instigated unfounded news reports about human toxicity of the dietary supplement L-norvaline. Most importantly, the study at hand does not confirm any human toxicity of L-norvaline; however, it makes claims unsupported by actual data, which resonate in newspaper articles and interviews. For example, they claim that “Bodybuilding supplement could be bad for the brain”, which is a misleading and false statement.

## References

[1] Samardzic K., Rodgers K.J. (2019). Cytotoxicity and mitochondrial dysfunction caused by the dietary supplement l-norvaline. Toxicol. In Vitro.

[2] Eagle H. (1955). The specific amino acid requirements of a human carcinoma cell (strain hela) in tissue culture. J. Exp. Med..

[3] Da Luz Dias R., Basso B., Donadio M.V.F., Pujol F.V., Bartrons R., Haute G.V., Gassen R.B., Bregolin H.D., Krause G., Viau C. (2018). Leucine reduces the proliferation of MC3T3-E1 cells through DNA damage and cell senescence. Toxicol. In Vitro.

[4] El-Bassossy H.M., El-Fawal R., Fahmy A., Watson M.L. (2013). Arginase inhibition alleviates hypertension in the metabolic syndrome. Br. J. Pharmacol..

[5] Polis B., Srikanth K.D., Elliott E., Gil-Henn H., Samson A.O. (2018). L-Norvaline Reverses Cognitive Decline and Synaptic Loss in a Murine Model of Alzheimer’s Disease. Neurotherapeutics.

[6] Ming X.F., Rajapakse A.G., Carvas J.M., Ruffieux J., Yang Z. (2009). Inhibition of S6K1 accounts partially for the anti-inflammatory effects of the arginase inhibitor L-norvaline. BMC Cardiovasc. Disord..

[7] Davoodi J., Drown P.M., Bledsoe R.K., Wallin R., Reinhart G.D., Hutson S.M. (1998). Overexpression and characterization of the human mitochondrial and cytosolic branched-chain aminotransferases. J. Biol. Chem..

[8] Schousboe A., Waagepetersen H.S. (2005). Role of astrocytes in glutamate homeostasis: Implications for excitotoxicity. Neurotox. Res..

[9] Rose C.R., Felix L., Zeug A., Dietrich D., Reiner A., Henneberger C. (2018). Astroglial glutamate signaling and uptake in the hippocampus. Front. Mol. Neurosci..

[10] Ormel L., Stensrud M.J., Chaudhry F.A., Gundersen V. (2012). A distinct set of synaptic-like microvesicles in atroglial cells contain VGLUT3. Glia.

[11] De Simone U., Caloni F., Gribaldo L., Coccini T. (2017). Human Co-culture model of neurons and astrocytes to test acute cytotoxicity of neurotoxic compounds. Int. J. Toxicol..

[12] Cynober L.A. (2003). Metabolic & Therapeutic Aspects of Amino Acids in Clinical Nutrition.

[13] Basun H., Forssell L.G., Almkvist O., Cowburn R.F., Eklöf R., Winblad B., Wetterberg L. (1990). Amino acid concentrations in cerebrospinal fluid and plasma in Alzheimer’s disease and healthy control subjects. J. Neural Transm. Park Dis. Dement. Sect..

[14] Tews J.K., Harper A.E. (1986). Tissue amino acids in rats fed norleucine, norvaline, homoarginine or other amino acid analogues. J. Nutr..

